# Intestinal parasites among migrant barn swallows (*Hirundo rustica*) in the central region of Mazandaran Province, Northern Iran

**DOI:** 10.14202/vetworld.2018.1179-1182

**Published:** 2018-08-28

**Authors:** Mahdi Fakhar, Tooran Nayeri Chegeni, Reza Bastani, Zahra Hosseininejad, Reza Saberi, Saber Armat

**Affiliations:** 1 Molecular and Cell Biology Research Center, Department of Parasitology, Faculty of Medicine, Mazandaran University of Medical Sciences, Sari, Iran; 2 Student Research Committee, Department of Parasitology, Faculty of Medicine, Mazandaran University of Medical Sciences, Sari, Iran

**Keywords:** barn swallow, *Hirundo rustica*, intestinal parasites, Iran, zoonosis

## Abstract

**Aim:**

Swallows are a family of migratory birds found worldwide except Antarctica. Annually, a number of species of swallows migrate to Iran. As they make their nests close to human living places, this may be a potential risk for public health. Conversely, no study has been conducted on intestinal parasitic infections of these birds so far. Therefore, the objective of this study was to determine the prevalence of intestinal parasites in migratory swallows (*Hirundo rustica*) in the central region of Mazandaran Province, Northern Iran.

**Materials and Methods:**

In this cross-sectional study, 205 feces samples from two districts (Sari and Qaemshahr) in the central region of Mazandaran were randomly collected during the summer and spring sessions of 2016-2017. The collected samples were examined using the routine direct fecal examination and formalin-ethyl acetate concentration. In addition, the samples were examined by cold acid-fast staining method to detect possible *Cryptosporidium* oocysts.

**Results:**

The results of this study indicated that 38 samples (18.5%) were infected with intestinal parasites. Among the helminthic parasites, eight genera and species were identified as follows: *Ascaridia galli*, *Syngamus trachea*, *Raillietina*, *Toxocara* spp., *Choanotaenia*, *Taenia* spp., *Ascaridia* spp., and *Moniezia* spp. In addition, among protozoan parasites, only the *Coccidia* spp. oocysts were identified.

**Conclusion:**

Our findings showed a relatively high prevalence of parasitic infections in migratory barn swallows in Mazandaran Province. Given the presence of zoonotic parasites in the samples, further investigations are needed to identify all parasites fauna, particularly zoonotic species among swallows in the region.

## Introduction

Swallows are a family of passerine birds (Hirundinidae family) found around the world. There are around 83 species and 19 genera [1]. These migratory birds are found around the world on all continents except Antarctica. The barn swallow, *Hirundo rustica*, is the most widespread species of swallow in the world. There are six subspecies of *H. rustica*, which breed across the Northern Hemisphere. Of these species, four are migratory birds among which *H. rustica* migrates to Northern Iran in spring. These birds can live in different geographic locations. However, these birds feed on insects; they often live in places where insects are abundantly found [1-3].

Swallows are closely related to people’s lives in both urban and rural residential houses. Swallows can easily nest in residential places, and most of them return to their nests each year and they may prefer to choose the same nest [2,4]. According to the annual censuses, about 2 million migratory birds come to Iranian seashores and wetlands every year. On the other hand, studies have shown that birds can transmit a variety of potential pathogens to pets and humans including *Cryptosporidium*, *Capillaria, Heterakis gallinarum, Syngamus*, and *Cryptococcus* [5-8].

Common zoonotic parasitic diseases are one of the major health, economic, and social problems in many developing countries including Iran. The transition of these diseases to humans is an important issue in terms of health and veterinary medicine, especially for the health-care system in providing services and against common infectious diseases. Our previous study in Mazandaran Province (area of the present study) showed that excreta of swallow harbor various species of yeasts, mainly *Cryptococcus neoformans* [9].

Our literature review showed that no study has been conducted on the prevalence of intestinal parasitic infections of these birds in the world as well as Iran. Therefore, the present study was conducted with the aim of investigating the parasitic fauna and assessing its potential in infecting the peripheral environment in Mazandaran Province.

## Materials and Methods

### Ethical approval

During all stages of our research, all applicable international, national, and/or institutional guidelines for the care and use of animals were followed. The fecal samples were collected according to the ethical approval of the Student Research Committee at Mazandaran University of Medical Sciences.

### Study area

Throughout the summer and spring sessions of 2016-2017, the present study was carried out in two districts, Sari (as capital of Mazandaran Province) and Qaemshahr (as one of the main cities) in the central region of Mazandaran Province (53°6’ E, 36°23’ N), Northern Iran. This province has a humid and temperate weather conditions with an average yearly rainfall of about 1000-1200 mm [10].

### Sample collection

To investigate intestinal parasite infection in swallows of Sari and Qaemshahr, 205 samples were collected by cluster sampling. The samples were randomly collected from different regions of these two cities (streets, residential houses, hospitals, and public places). The date of sampling and related address was written on each container for any probable resampling purpose. For sampling, the nests of the swallows in different areas of the cities were identified, and the feces were collected right from their nests. Immediately after collection, the feces samples were transferred to the parasitology laboratory of Mazandaran University of Medical Sciences.

### Sample examination and identification

All samples were first examined by routine direct fecal examination method. Then, formalin-ethyl acetate technique using Parasite test kit (Paramed, Iran) was used to detect possible parasite eggs and cysts as follows: The two matching parts of the test tube were unscrewed, and the cylindrical part was placed inside the tube. Then, 3.5 mL of 10% formalin-saline and ethyl-acetate working solution was added, and some feces (about 1 G) were taken by abaisse-langue and added to the solution. The tube was well vortexed for 30 s, in a way that the cone section was upright. In the next step, the parasite test was reversed and centrifuged at 400 g for 3 min. After centrifugation, the tube was abruptly opened, and the solution in a cylindrical part was discarded. Then, the remaining fluid in the conical portion was taken, the upper part was slightly removed, the residual was mixed. At the final stage, a drop of sediment was placed on the slide and examined under the microscope in terms of parasite eggs and cysts. In addition, to investigate the presence of *Cryptosporidium* spp., in the samples taken from the swallows, cold acid-fast staining method was used [11]. Finally, morphological characteristics were used for every specimen to detect the identified parasites based on identification key [12]. Our study was a descriptive cross-sectional study; hence, a negative control was not included in the study.

## Results

**Figure-1:**
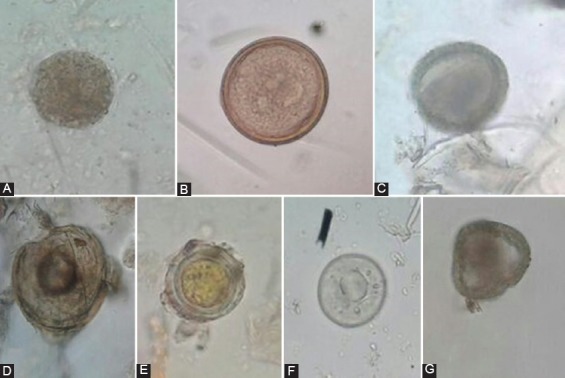
Images showing intestinal parasites identified in immigrant barn swallows feces in Mazandaran Province (40×). (a) *Ascaridia galli*, (b) *Taenia* spp., (c) *Toxocara* spp. egg, (d) *Raillietina* spp., (e) *Choanotaenia* spp., (f) *Coccidia* oocyst, (g) *Moniezia* spp.

In this study, 205 samples of swallow feces were examined for parasitic infections, of which 38 samples (38/205, 18.5%) were positive. The worm infections belong to eight species and genera, including *Ascaridia* *gall*, *Toxocara* spp., *Taenia* spp., *Syngamus trachea*, *Raillietina* spp., *Moniezia* spp., *Ascaridia* spp., and *Choanotaenia* spp. Furthermore, among protozoan infections, *Coccidia* oocytes were the only infection detected (Figure-1). The highest prevalence in the infected swallows was related to *A. galli* (20/38, 52.3%) and then *Taenia* spp. (6/38, 15.8%) (Table-1). Furthermore, no trematode was found in the samples examined. It should also be noted that no cases of *Cryptosporidium* infection were detected in this study. Meanwhile, of the total of 38 positive specimens, 22 (57.9%) belonged to Qaemshahr and 16 (42.1%) to Sari districts.

**Table-1:** Prevalence of intestinal parasites in feces of migratory swallows according to parasite species and place in Mazandaran Province.

Parasite species	City	Number of positive (%)
*Ascaridia galli*	Qaemshahr	20 (52.3)
*Taenia* spp.	Sari	6 (15.8)
*Toxocara* spp.	Sari	5 (13.1)
*Syngamus trachea*	Sari	2 (5.3)
*Raillietina* spp.	Sari	1 (2.7)
*Moniezia* spp.	Sari	1 (2.7)
*Choanotaenia* spp.	Sari	1 (2.7)
*Ascaridia* spp.	Qaemshahr	1 (2.7)
*Coccidia* spp. oocyst	Qaemshahr	1 (2.7)
Total		38 (18.5)

## Discussion

The results of our preliminary study indicate a relatively high prevalence (18.5%) of parasitic infections among migratory swallows of Mazandaran Province. The highest infection rates were related to *A. galli*, *Taenia* spp., and *Toxocara* spp. with 52.3%, 15.8%, and 13.1%, respectively. As it was mentioned, no study has investigated the parasitic infection of migratory swallows in Iran; though, several studies have been conducted on hemoparasites such as *Plasmodium* spp., and ectoparasites of these birds in other regions of the world [13]. The high infection rate of *A. galli* in our study is almost similar to the results of Eslami *et al*. [14] that reported a high prevalence (56%) of *A. galli* among native free-range fowls in the Golestan Province, Northern Iran. They also reported other species of parasites which detected in alimentary canals of these birds as follows: *H. gallinarum* (24%), *Capillaria anatis* (4%), *Cheilospirura hamulosa* (4%), *Raillietina tetragona* (58%), *Raillietina echinobothrida* (6%), and *Choanotaenia infundibulum* (8%); lungs: *S. trachea* (16%) [14].

Among parasitic infections of the birds, *Choanotaenia* spp. infects the posterior part of the small intestine of poultries and turkeys, and *Raillietina* spp. can involve the posterior part of the small intestine of the poultries, guinea fowls, turkeys, pheasants, and quails. These worm parasites have also been reported from different types of birds in Iran [14,15]. In addition, *Ascaridia* spp. and *S. trachea* have been reported in the domestic and wild poultry of Iran [14-18]. However, the most studies focused on intestinal, blood parasites, and recently avian schistosomes in native and not often in migratory bird species in Iran [14-21].

In this study, 11 samples were infected with *Taenia* spp. and *Toxocara* spp.; both of which have the potential risk for infecting the human population. Given that swallows usually nest in residential places such as homes and hospitals, and because of the transmission of zoonotic parasitic infections by these birds, it is believed that swallows can pose a serious threat to human health and environment [22].

Furthermore, a *Moniezia* spp. (a parasite of ruminants) and *Toxocara* spp. (a parasite of dogs and cats) eggs were found in the swallow excreta. Swallows use wood and leaves to build their nests, which are likely to be infected to ruminants’ and carnivores fecal materials. It seems that the swallowing of the feces by these birds has caused infection by *Moniezia* and *Toxocara* spp. parasites. Consequently, those harbored these parasites considered as pseudo host, because the parasites transiently exist within the alimentary canal.

Our data demonstrate that fecal samples of swallows may harbor different species of parasites (mainly *A. galli*, *Taenia* spp., and *Toxocara* spp.) and may be capable of distributing these parasites in the private and public environments. Considering the fact that migratory swallows are common in Iran and having a coexisting relationship with humans in our country; therefore, it needs an awareness of the general population to prevent transmission of the parasitic infections by barn swallow in the region.

## Conclusion

Given that the migratory swallows are considered as a holy bird in Iran and build their nests in closeness to human homes and around the public places, for example, hospitals and streets, these birds might be as a latent and hazardous source for distribution of zoonotic parasitic infections. As a whole, our results also highlight further studies on the distribution of gastrointestinal parasites among swallow excreta, especially zoonotic ones and determination true burden of these in other parts of Iran as well as the worldwide.

## Authors’ Contributions

MF designed all steps of the study, RB and SA collected the samples, ZH and TNC examined the collected samples, and RS wrote the manuscript draft. All authors read, revised, and approved the final draft.
